# Surface Morphology Control and Amino Functionalization of Silicon Nanoparticles for Enhanced Antibody Immobilization

**DOI:** 10.3390/ma19194069

**Published:** 2026-09-23

**Authors:** Myat Endra Swe, Yoshinobu Manome, Akinori Ueno, Keisuke Sato

**Affiliations:** 1 Department of Electrical and Electronic Engineering, Tokyo Denki University, 5 Senju-Asahi-cho, Adachi-ku, Tokyo 120-8551, Japan; ueno@mail.dendai.ac.jp (A.U.); satokeisuke2010@gmail.com (K.S.); 2 Core Research Facilities, Jikei University School of Medicine, 3-25-8 Nishi-Shinbashi, Minato-ku, Tokyo 105-8461, Japan; yoshi.manome@gmail.com

**Keywords:** silicon nanoparticles, metal-assisted chemical etching, specific surface area, amino functionalization, antibody immobilization, immunoassay

## Abstract

The large specific surface area of nanoparticles makes them an attractive platform for antibody immobilization in immunoassays, but the combined effects of their surface structuring and chemical functionalization on properties relevant to antibody immobilization have not yet been clarified. Here, porous and spiky silicon nanoparticles (SiNPs) were fabricated by metal-assisted chemical etching, and the relationships among specific surface area, amino functionalization, and the JT-95-related ELISA signal following BS3-mediated immobilization were investigated. The formation of the porous and spiky surface structures was confirmed by scanning electron microscopy, including quantitative image analysis, and nitrogen adsorption analysis revealed that the specific surface area increased from 62.3 m^2^ g^−1^ for pristine SiNPs to 111.8 and 142.2 m^2^ g^−1^ for porous and spiky SiNPs, respectively. Fourier transform infrared spectroscopy and the ninhydrin assay confirmed successful amino functionalization, and the ninhydrin response increased with increasing specific surface area. An enzyme-linked immunoassay further demonstrated that the JT-95-related ELISA signal was significantly higher for amino-functionalized porous and spiky SiNPs than for amino-functionalized pristine SiNPs (*p* < 0.01). Although the mean ELISA signal was higher for spiky SiNPs than for porous SiNPs, the difference was not statistically significant. Overall, these results strongly suggest an association among increased specific surface area, enhanced amino functionalization, and increased JT-95-related ELISA signal. These findings provide useful design guidelines for SiNP-based antibody immobilization platforms and their future application in immunoassays and biosensing.

## 1. Introduction

Immunoassays are a widely used analytical technique for diagnosing diseases and detecting biomarkers. Enzyme-linked immunosorbent assays (ELISAs) are particularly prevalent because of their high specificity and simplicity, which make them indispensable for both clinical diagnostics and biomedical research [1,2,3]. However, conventional ELISAs rely on immobilizing antibodies on solid-phase substrates such as microtiter plates, which inherently limits the available surface area for antibody immobilization. In addition, physical adsorption-based immobilization often suffers from antibody desorption and random orientation, which reduces the stability and reproducibility of immunoassays [1,4].

To overcome these limitations, nanoparticles have attracted considerable attention owing to their large specific surface area, which potentially increases the surface available for antibody immobilization and makes nanoparticles attractive as solid-phase platforms for immunoassays. However, antibodies are still commonly immobilized on nanoparticles by physical adsorption, which limits immunoassay stability and reproducibility [1,5]. Although covalent immobilization strategies have been developed, the relationships between nanoparticle surface morphology, chemical functionalization, and surface properties relevant to antibody immobilization have not yet been fully clarified [5].

Silicon nanoparticles (SiNPs) have been extensively investigated as biomaterials because of their excellent biocompatibility and ease of surface modification [6,7,8]. Metal-assisted chemical etching (MACE) has been shown to be capable of fabricating diverse surface morphologies on silicon surfaces [9,10]. Therefore, surface structuring of SiNPs by MACE and the associated changes in specific surface area may influence surface properties relevant to antibody immobilization.

In this study, porous silicon nanoparticles (SiNPPs) and spiky silicon nanoparticles (SiNKPs) were fabricated by MACE, and the particle surfaces were subsequently functionalized with amino groups, followed by BS3-mediated covalent immobilization of JT-95. The relationships among specific surface area, APTES-mediated amino functionalization, and the JT-95-related ELISA signal were evaluated in the surface-structured SiNPs.

## 2. Materials and Methods

### 2.1. Materials

SiNPs with an average diameter of approximately 100 nm (according to manufacturer specifications) were purchased from Thermo Fisher Scientific (Waltham, MA, USA). Ethanol, 95% sulfuric acid (H_2_SO_4_), 60% hydrogen peroxide (H_2_O_2_), 46% hydrofluoric acid (HF), and 60% nitric acid (HNO_3_) were obtained from Wako Pure Chemical Industries (Osaka, Japan). Silver nitrate (AgNO_3_) was purchased from Sigma-Aldrich (St. Louis, MO, USA), and 3-aminopropyltriethoxysilane (APTES) was obtained from Shin-Etsu Silicones (Tokyo, Japan). Ninhydrin reagent, phenol, tin (II) chloride dihydrate, and pyridine were obtained from Nacalai Tesque (Kyoto, Japan). Bis(sulfosuccinimidyl)suberate (BS3) was obtained from Dojindo (Kumamoto, Japan). Phosphate-buffered saline (PBS) and PBS-T were obtained from MP Biomedicals (Irvine, CA, USA) and Takara Bio (Shiga, Japan), respectively. Hybridoma cell culture medium (COSMEDIUM 005) was obtained from Cosmo Bio (Tokyo, Japan). A blocking reagent (Block Ace) was obtained from KAC (Kyoto, Japan). IgM-Po (goat anti-mouse IgM-HRP) was purchased from Santa Cruz Biotechnology (Dallas, TX, USA), and 3,3′,5,5′-tetramethylbenzidine (TMB) substrate was obtained from BD Biosciences (Franklin Lakes, NJ, USA). JT-95-producing hybridoma cells were provided by a collaborator and cultured in our laboratory to obtain JT-95-containing hybridoma culture medium for antibody immobilization. All chemicals were used as received without further purification.

### 2.2. Surface Structuring of Silicon Nanoparticles

MACE was performed to structure the surfaces of the SiNPs [9,10]. Initially, 300 mg of SiNPs was dispersed in deionized water and ultrasonicated for 5 min using an ultrasonic homogenizer (Branson 450D Advanced Digital Sonifier, Branson Ultrasonics Corporation, Danbury, CT, USA; frequency: 20 kHz, power: 400 W, amplitude: 30%, ON/OFF: 10 s/5 s) to obtain a homogeneous suspension. An etching solution containing HF and AgNO_3_ was then added to the suspension, followed by stirring at 50 rpm for approximately 1 min at room temperature (25–28 °C) to deposit Ag nanoparticles onto the SiNP surfaces. Subsequently, H_2_O_2_ was added, and the mixture was stirred at 50 °C and 50 rpm for 10 min to promote the etching reaction. During this process, the Ag nanoparticles acted as catalysts for the localized oxidation and dissolution of silicon, thereby promoting surface structuring of the SiNPs. Among the MACE conditions, the AgNO_3_ concentration was varied between the two surface-structured particle types: 0.01 M for SiNPPs and 0.02 M for SiNKPs, whereas the HF concentration (3.0 M), H_2_O_2_ concentration (0.06 M), reaction temperature (50 °C), stirring speed (50 rpm), etching time (10 min), and final MACE solution volume (50 mL) were kept constant. The detailed MACE etching conditions used for the preparation of SiNPPs and SiNKPs are summarized in Table 1.

After etching, the particles were collected by filtration and rinsed with more than 100 mL of deionized water. The particles were then immersed in more than 100 mL of deionized water, ultrasonicated for 10 min, and collected by filtration; this washing procedure was repeated three times. Subsequently, the particle-containing filter was immersed in commercially available concentrated HNO_3_ for 20 min. After the HNO_3_ treatment, the particles were collected by filtration and subjected to the same washing procedure with deionized water at least three times. The washed particles were dried on a hotplate at ≥80 °C for at least 20 min and stored until further surface treatment.

The morphology of the prepared SiNPPs and SiNKPs, along with pristine SiNPs used as the control, was observed by field-emission scanning electron microscopy (FE-SEM, Hitachi Regulus 8100, Hitachi, Tokyo, Japan) at an acceleration voltage of 10 kV. For quantitative morphological evaluation, image analysis was additionally performed using five different SEM images for each particle type. ImageJ 1.54g software was calibrated using the scale bar in each SEM image. For SiNPs, SiNPPs, and SiNKPs, one particle was selected from each SEM image, and the particle contour was traced to determine the Feret and MinFeret diameters (*n* = 5 particles per particle type). The particle size distribution, median diameter, cumulant diameter (Z-average), and polydispersity index (PDI) in deionized water were determined by dynamic light scattering (DLS, HORIBA SZ-100HS2, HORIBA, Ltd., Kyoto, Japan) at room temperature (25–28 °C). Nitrogen adsorption–desorption measurements were performed using a MicrotracBEL Belsorp-mini II analyzer (MicrotracBEL Corp., Osaka, Japan) at 77 K over a relative pressure range of 0.02–0.99 (P/P0). The specific surface area was calculated using the Brunauer–Emmett–Teller (BET) method. For both DLS and BET analyses, one measurement was performed for each particle type using the corresponding particle batch (*n* = 1 per particle type).

### 2.3. Amino Functionalization of Silicon Nanoparticles

For amino functionalization of the SiNP surfaces, the particles were hydroxylated and subsequently amino-functionalized using APTES [11,12,13,14]. Prior to hydroxylation, the particles were dispersed in deionized water and ultrasonicated for 5 min using an ultrasonic homogenizer (Branson 450D Advanced Digital Sonifier, Branson Ultrasonics Corporation, Danbury, CT, USA; frequency: 20 kHz, power: 400 W, amplitude: 30%, ON/OFF: 10 s/5 s). HF was then added to obtain a deionized water/HF ratio of 9.5/0.5 (*v*/*v*), and the particles were treated for 4 min to remove the surface oxide layer. The particles were subsequently collected by filtration and washed three times with deionized water.

Pristine SiNPs, SiNPPs, and SiNKPs were first treated with a piranha solution (H_2_SO_4_/H_2_O_2_ = 3:1, *v*/*v*) at 70 °C while being stirred at 50 rpm for 50 min to introduce hydroxyl groups onto the particle surfaces. The resulting hydroxylated particles are hereby referred to by the prefix “OH-” (i.e., OH-SiNPs, OH-SiNPPs, and OH-SiNKPs). After a thorough rinsing with deionized water, the hydroxylated particles were dispersed in a solution comprising 1 mL of APTES and 49 mL of deionized water and were allowed to react at 40 °C under stirring at 300 rpm for 30 min. The samples were subsequently heated on a hotplate at 80 °C for 20 min to promote the condensation reaction between APTES and the hydroxyl groups on the particle surfaces. The resulting amino-functionalized particles are hereby referred to by the prefix “NH-” (i.e., NH-SiNPs, NH-SiNPPs, and NH-SiNKPs).

The amino functionalization of the nanoparticle surfaces was confirmed by Fourier transform infrared (FTIR) spectroscopy (JASCO FT/IR-4600, JASCO Corporation, Tokyo, Japan) at room temperature (25–28 °C) [12,13]. The introduced surface amino groups were evaluated using the ninhydrin assay [15]. Briefly, 3 mg of OH-SiNPs, NH-SiNPs, NH-SiNPPs, or NH-SiNKPs was placed in a test tube. Subsequently, 50 µL of ninhydrin solution (50 mg/mL in ethanol), 50 µL of phenol solution (80 mg phenol dissolved in 20 mL ethanol), and 50 µL of 0.01 M tin (II) chloride dihydrate dissolved in pyridine were added. Each mixture was heated at 100 °C for 5 min. After the reaction, the mixtures were diluted with 5 mL of 70% ethanol. Subsequently, 100 µL of each sample was transferred to a 96-well microplate, and the absorbance at 570 nm was measured using a microplate reader. The absorbance values were normalized to 1 mg of particles and compared among the groups to evaluate the relative amounts of amino groups introduced onto the nanoparticle surfaces. The ninhydrin assay was performed in five independent experiments (*n* = 5), and the data are presented as the mean ± standard deviation (SD). Differences among the four groups were analyzed by one-way analysis of variance (ANOVA), followed by the Tukey–Kramer multiple-comparison test. A *p*-value of <0.05 was considered statistically significant.

### 2.4. Antibody Immobilization onto Silicon Nanoparticles

JT-95 is a mouse monoclonal IgM antibody that specifically reacts with papillary thyroid carcinoma [16,17]. IgM antibodies characteristically exhibit a relatively large pentameric structure [18,19]. BS3 was employed as an amino-reactive crosslinker, and a crosslinking solution was prepared by dissolving 5 mg of BS3 in 87 µL of 100 mmol/L phosphate-buffered saline (PBS).

Antibody immobilization was performed as follows. Briefly, 10 mg of NH-SiNPs, NH-SiNPPs, or NH-SiNKPs was placed into a 1.5 mL microcentrifuge tube, followed by the addition of 300 µL of a hybridoma culture medium containing JT-95 antibodies with a total protein concentration of 2.2 mg/mL as determined by spectrophotometry (NanoDrop OneC, Thermo Fisher Scientific). The suspension was then briefly mixed using a vortex mixer for 2–3 s. Subsequently, 10 µL of the prepared crosslinking solution was added, and the microcentrifuge tubes were placed horizontally on a tube rotator and gently rotated for 10 min. This procedure was repeated four times in total. After the first three additions, the samples were rotated for 10 min each. After the fourth addition, the samples were rotated for a further 30 min to promote covalent coupling between the amino-functionalized nanoparticle surfaces and proteins in the JT-95-containing hybridoma culture medium. After immobilization was completed, the samples were stored at 4 °C until subsequent immunoassay experiments.

### 2.5. Evaluation of JT-95-Related ELISA Signal

The JT-95-related signal from the SiNP surfaces was evaluated through an immunoassay based on an HRP-conjugated secondary antibody. NH-SiNPs, NH-SiNPPs, and NH-SiNKPs stored at 4 °C were first collected by centrifugation at 15,000 rpm and 4 °C for 2 min, and the supernatant was discarded. OH-SiNPs, which were not subjected to antibody immobilization, were included as a blank control to evaluate the background signal of the immunoassay.

The following procedure was performed simultaneously for all four groups (i.e., OH-SiNPs, NH-SiNPs, NH-SiNPPs, and NH-SiNKPs). Samples were washed with PBS by centrifugation at 15,000 rpm and 4 °C for 2 min, and this washing procedure was repeated four times. During each washing procedure, the particle suspensions were briefly redispersed using a vortex mixer for 2–3 s before centrifugation. To suppress nonspecific adsorption, 100 µL of Block Ace was added to each sample, followed by incubation at 4 °C for more than 1 h [20]. After blocking, the blocking solution was removed. The particles were then washed with PBS-T by centrifugation at 15,000 rpm and 4 °C for 2 min, and this washing procedure was repeated four times. Subsequently, 80 µL of IgM-Po (goat anti-mouse IgM-HRP) diluted 500-fold with PBS-T was added, and the samples were incubated for 1 h. After incubation, the particles were washed with PBS-T by centrifugation at 15,000 rpm and 4 °C for 2 min, which was repeated four times, to remove unbound secondary antibodies. During each PBS-T washing procedure, the particle suspensions were briefly redispersed using a vortex mixer for 2–3 s before centrifugation. Next, 60 µL of TMB substrate solution was added, and the samples were incubated in the dark at room temperature (25–28 °C) for approximately 10 min [21,22]. The enzymatic reaction was then terminated by adding 60 µL of an H_2_SO_4_-based stop solution. Subsequently, 100 µL of the reacted solution from each sample was transferred to a 96-well microplate, and the absorbance at 450 nm was measured using a microplate reader. The obtained absorbance values were normalized to 1 mg of particles and compared among the groups as the JT-95-related ELISA signal. All four groups were measured simultaneously on the same microplate.

The immunoassay described above was independently repeated on five different days (*n* = 5). Statistical analysis was performed using JMP Student Edition19.0.5 (JMP Statistical Discovery LLC., Cary, NC, USA). Differences among the four groups were analyzed by one-way ANOVA. When significant differences were detected, Tukey–Kramer’s honestly significant difference test was applied for multiple comparisons. Statistical significance was defined as *p* < 0.05, and the results are presented as the mean ± SD.

### 2.6. Relationship Analyses

To evaluate the relationships among BET specific surface area, amino functionalization, and the JT-95-related ELISA signal, the ninhydrin and ELISA results were descriptively compared with the BET specific surface area of each particle type. The relationships between BET specific surface area and ninhydrin absorbance at 570 nm and between BET specific surface area and the JT-95-related ELISA signal at 450 nm were visualized using the five independent measurements obtained for each particle type.

Because the BET measurement was performed once for each particle type (*n* = 1 per particle type), inferential correlation analysis was not performed using the individual ninhydrin or ELISA measurements. Instead, the mean ninhydrin absorbance and mean JT-95-related ELISA signal for each particle type were plotted against the corresponding BET specific surface area, and linear trend lines were fitted to the mean values of the three particle types to descriptively evaluate the overall trends.

In addition, to compare the ninhydrin response while accounting for differences in specific surface area, the BET-normalized ninhydrin response ($R_{BET}$) was calculated using Equation (1), as follows:

(1) $$R_{BET}=\frac{A_{570\mathrm{nm},\;1\mathrm{mg}}}{S_{BET}\times 0.001}$$

where $A_{570\mathrm{nm},\;1\mathrm{mg}}$ represents the mean ninhydrin absorbance at 570 nm normalized to 1 mg of particles, and $S_{BET}$ represents the BET specific surface area [m^2^/g] of each particle type. The BET-normalized ninhydrin response was calculated for NH-SiNPs, NH-SiNPPs, and NH-SiNKPs.

## 3. Results

### 3.1. Characterization

Figure 1 shows the surface morphologies and particle size distributions of the prepared SiNPs obtained by SEM and DLS. Pristine SiNPs (Figure 1a) exhibited a spherical morphology with smooth surfaces. SiNPPs (Figure 1c) exhibited numerous fine pore-like structures over their surfaces, whereas SiNKPs (Figure 1e) exhibited spiky protrusions distributed over their surfaces. Quantitative morphological analysis was performed using multiple SEM images, and the results are summarized in Table 2. The DLS measurements showed median particle diameters of 259.3, 186.3, and 196.2 nm for SiNPs, SiNPPs, and SiNKPs, respectively (Figure 1b,d,f). The corresponding cumulant diameters (Z-average) were 258.5, 187.4, and 196.7 nm, respectively, with PDI values of 0.281, 0.191, and 0.254, respectively.

Figure 2 shows the nitrogen adsorption–desorption isotherms of the prepared SiNPs. Both SiNPPs and SiNKPs exhibited typical Type IV nitrogen adsorption–desorption isotherms accompanied by hysteresis loops that became apparent at relative pressures (P/P_0_) above approximately 0.45 [23]. In contrast, pristine SiNPs exhibited only a negligible hysteresis loop. Table 3 presents the corresponding specific surface areas. BET analysis showed that the specific surface area increased from 62.3 m^2^ g^−1^ for pristine SiNPs to 111.8 and 142.2 m^2^ g^−1^ for SiNPPs and SiNKPs, respectively.

### 3.2. Amino Functionalization

As shown in Figure 3, the amino-functionalized SiNPs exhibited characteristic absorption bands corresponding to Si–O–Si, CH_2_, and NH_2_ groups. A strong absorption band at approximately 1130–1200 cm^−1^ was assigned to the asymmetric stretching vibration of Si–O–Si bonds [24]. Absorption bands observed at 1484 and 1562 cm^−1^ were attributed to NH_2_ deformation vibrations, those at 2883 and 2932 cm^−1^ were attributed to CH_2_ stretching vibrations, and the broad band around 3370 cm^−1^ was attributed to N–H stretching vibrations [12,13,24]. In contrast, OH-SiNPs without amino functionalization showed no characteristic absorption bands corresponding to CH_2_ or NH_2_ groups, and only the Si–O–Si absorption band was observed. Furthermore, no characteristic absorption bands attributable to the ethoxy groups of APTES, such as CH_3_ or Si–O–C vibrations, were detected. Figure 4 shows the results of the ninhydrin assay. NH-SiNPs, NH-SiNPPs, and NH-SiNKPs exhibited a distinct bluish-purple coloration, whereas OH-SiNPs remained nearly colorless. Figure 5 shows the ninhydrin absorbance at 570 nm normalized to 1 mg of particles, obtained from five independent experiments (*n* = 5) and expressed as the mean ± standard deviation (SD). The absorbance values for OH-SiNPs, NH-SiNPs, NH-SiNPPs, and NH-SiNKPs were 0.05 ± 0.02, 0.16 ± 0.02, 0.37 ± 0.08, and 0.62 ± 0.15, respectively. Tukey–Kramer multiple-comparison analysis showed that NH-SiNKPs exhibited significantly higher absorbance than OH-SiNPs (*p* < 0.0001), NH-SiNPs (*p* < 0.0001), and NH-SiNPPs (*p* = 0.0011). NH-SiNPPs also exhibited significantly higher absorbance than OH-SiNPs (*p* = 0.0001) and NH-SiNPs (*p* = 0.0071). In contrast, no statistically significant difference was observed between NH-SiNPs and OH-SiNPs (*p* = 0.2251).

### 3.3. JT-95-Related ELISA Signal

The JT-95-related ELISA signal of the prepared SiNPs was evaluated using an HRP-conjugated secondary antibody. Figure 6 shows the color development after the enzymatic reaction. Little or no color development was observed for OH-SiNPs. In contrast, NH-SiNPs, NH-SiNPPs, and NH-SiNKPs exhibited distinct color development after the enzymatic reaction.

Figure 7 shows the measured absorbance at 450 nm normalized to 1 mg of particles, which was used to compare the JT-95-related ELISA signals among the four groups. NH-SiNPPs and NH-SiNKPs exhibited significantly higher absorbance values than NH-SiNPs (*p* = 0.0052 and *p* < 0.0001, respectively). Among the four groups, NH-SiNKPs exhibited the highest mean absorbance, followed by NH-SiNPPs, NH-SiNPs, and OH-SiNPs. Although the mean absorbance of NH-SiNKPs was higher than that of NH-SiNPPs, the difference was not statistically significant (*p* = 0.1487). Similarly, the mean absorbance of NH-SiNPs was higher than that of OH-SiNPs, although the difference was not statistically significant (*p* = 0.1051).

### 3.4. Relationship Between Specific Surface Area and Ninhydrin and JT-95-Related ELISA Signals

To evaluate the relationships among BET specific surface area, amino functionalization, and the JT-95-related ELISA signal, exploratory bivariate analyses were performed. Samples used for BET measurements, the ninhydrin assay, and ELISA measurements were obtained by subdividing the same particle batch prepared for each particle type. The BET specific surface area was measured once for each particle type (*n* = 1 per particle type), and the obtained value was used as the representative specific surface area for the corresponding particle type. In contrast, the ninhydrin assay and ELISA measurements were independently performed five times for each particle type (*n* = 5). Accordingly, in the exploratory analyses, the representative BET specific surface area for each particle type was associated with the five corresponding absorbance measurements obtained from samples subdivided from the same particle batch.

Figure 8 shows the relationship between BET specific surface area and ninhydrin absorbance at 570 nm normalized to 1 mg of particles. Ninhydrin absorbance showed an overall increasing trend with increasing BET specific surface area. Under the exploratory analysis described above, a positive association was observed between BET specific surface area and ninhydrin absorbance (r = 0.89, *p* < 0.0001). Because a single representative BET value was used for each particle type, these statistical values should be interpreted as descriptive of the observed particle-type trend rather than as evidence based on independent replicate BET measurements.

Figure 9 shows the relationship between BET specific surface area and the JT-95-related ELISA signal at 450 nm normalized to 1 mg of particles. The JT-95-related ELISA signal showed an overall increasing trend with increasing BET specific surface area. Under the exploratory analysis described above, a positive association was observed between BET specific surface area and the JT-95-related ELISA signal (r = 0.86, *p* < 0.0001). As with the ninhydrin analysis, these statistical values reflect the relationship obtained using a single representative BET value for each particle type and should therefore be interpreted with this limitation.

The ninhydrin assay and ELISA measurements were performed using separate samples subdivided from the same particle batch for each particle type; therefore, individual ninhydrin and ELISA measurements were not paired on a one-to-one basis. Their relationship was instead descriptively compared using the mean ninhydrin absorbance and mean JT-95-related ELISA signal for each particle type. As shown in Figure 10, the mean JT-95-related ELISA signal increased across the three particle types with increasing mean ninhydrin absorbance.

Furthermore, to compare the ninhydrin response while accounting for differences in BET specific surface area, the BET-normalized ninhydrin response ($R_{BET}$) was calculated using Equation (1).

The $R_{BET}$ values were 2.59, 3.28, and 4.37 for NH-SiNPs, NH-SiNPPs, and NH-SiNKPs, respectively. Thus, differences in the ninhydrin response among the three particle types remained after normalization by BET specific surface area.

## 4. Discussion

### 4.1. Surface Morphology and Specific Surface Area

In this section, the effects of MACE-induced surface structuring on particle morphology and specific surface area are discussed in terms of SEM morphological analysis, MACE conditions, particle size determined by DLS, possible particle aggregation, and surface accessibility represented by BET measurements.

SEM observations showed distinct surface morphologies among the three particle types, with pore-like structures observed on SiNPPs and spiky protrusions on SiNKPs. Quantitative analysis using multiple SEM images further provided Feret and MinFeret diameters for each particle type. Because the AgNO_3_ concentration was the principal MACE parameter varied between SiNPPs and SiNKPs, differences in Ag deposition and the resulting localized etching may have contributed to the formation of the different surface structures. After MACE, HNO_3_ treatment and repeated washing were performed as post-etching cleaning steps. Because residual Ag was not directly quantified after these treatments, the effects of MACE were evaluated based on the observed surface morphology and physicochemical properties of the resulting particles rather than on the amount of residual Ag. BET analysis showed that the specific surface area increased following MACE-induced surface structuring, with SiNPPs and SiNKPs exhibiting larger specific surface areas than pristine SiNPs. This result supports the effectiveness of MACE-induced surface structuring for increasing the N_2_-accessible surface area of the SiNPs.

The particle dimensions obtained from SEM image analysis did not show the same trend as the particle sizes determined by DLS. This discrepancy may be attributable to differences in the measurement principles and sample populations evaluated by the two methods. SEM measurements were performed on individual particles in the dry state and were therefore not directly affected by particle aggregation during measurement, whereas DLS determines the hydrodynamic size of particles in dispersion and may be influenced by aggregation. In addition, because the commercial SiNPs have a particle size distribution and the SEM analysis was based on a limited number of individually observed particles, the particles included in the SEM analysis may not fully represent the overall particle size distribution. The smaller DLS particle sizes observed for SiNPPs and SiNKPs than for pristine SiNPs may also partly reflect a reduction in particle dimensions associated with the etching process. Therefore, the differences between the SEM- and DLS-derived particle dimensions may reflect a combination of the different measurement principles, particle selection in SEM analysis, possible aggregation in dispersion, and changes in particle dimensions caused by etching. Because the aggregation state was not directly evaluated in this study, its contribution to the observed DLS particle sizes could not be quantitatively assessed.

Furthermore, the BET specific surface area determined by N_2_ adsorption at 77 K represents the surface accessible to N_2_ molecules and is not directly equivalent to the surface area accessible to larger molecules such as APTES or IgM. Therefore, the BET specific surface area was interpreted as an indicator of the overall change in specific surface area associated with MACE-induced surface structuring rather than as a direct measure of the surface area available for antibody immobilization.

### 4.2. Effect of Specific Surface Area on Amino Functionalization

In this section, the relationship between BET specific surface area and APTES-mediated amino functionalization is discussed based on the FTIR and ninhydrin assay results.

The FTIR and ninhydrin assay results confirmed amino functionalization after APTES treatment. Importantly, the ninhydrin response increased across the three amino-functionalized particle types with increasing BET specific surface area. This consistent trend supports an association between the increase in specific surface area induced by MACE surface structuring and enhanced APTES-mediated amino functionalization.

The BET-normalized ninhydrin response also increased in the order NH-SiNPs < NH-SiNPPs < NH-SiNKPs, indicating that differences in the ninhydrin response remained even after accounting for the differences in BET specific surface area. This finding suggests that the observed differences in amino functionalization may not be explained solely by the total specific surface area and that factors associated with the different surface morphologies may also contribute to amino functionalization.

Although the ninhydrin assay does not directly quantify the absolute amount or surface density of amino groups, it provides a useful relative measure of amino functionalization among particle types treated under the same conditions. Taken together, the FTIR and ninhydrin results support an association between increased specific surface area and enhanced APTES-mediated amino functionalization in the surface-structured SiNPs, while the BET-normalized results suggest that surface morphology-related factors, in addition to specific surface area, may contribute to the observed differences in ninhydrin response.

### 4.3. JT-95 Immobilization-Related ELISA Response

In this section, the JT-95-related ELISA signal obtained following BS3-mediated immobilization is discussed in relation to amino functionalization and the interpretation of the immunoassay used in this study.

NH-SiNPPs and NH-SiNKPs exhibited significantly higher JT-95-related ELISA signals than NH-SiNPs. This result, together with the increased ninhydrin responses observed for the surface-structured particles, supports an association between enhanced amino functionalization following MACE-induced surface structuring and an increased ELISA signal after BS3-mediated JT-95 immobilization.

Although NH-SiNKPs exhibited a significantly higher ninhydrin response than NH-SiNPPs, the mean JT-95-related ELISA signal of NH-SiNKPs was higher than that of NH-SiNPPs without reaching statistical significance. This finding suggests that an increase in ninhydrin-detectable amino groups does not necessarily result in a proportional increase in the JT-95-related ELISA signal. Because IgM has a relatively large pentameric structure, accessibility of IgM to amino-functionalized sites on the structured particle surfaces may differ from the accessibility of the smaller molecules involved in APTES functionalization and the ninhydrin reaction. In addition, the spatial arrangement and accessibility of immobilized IgM to the secondary antibody may influence the resulting ELISA signal. These factors may partly explain why the difference between NH-SiNPPs and NH-SiNKPs observed in the ninhydrin assay was not reflected as a statistically significant difference in the ELISA signal.

Because JT-95-containing hybridoma culture medium was used for immobilization, proteins other than JT-95 present in the culture medium may also have reacted with BS3 and become immobilized on the particle surfaces. Nevertheless, the HRP-conjugated goat anti-mouse IgM antibody used for detection enabled measurement of signals derived from particle-associated mouse IgM, including JT-95. Therefore, the absorbance obtained in this study was interpreted as a JT-95-related ELISA signal rather than as a direct quantitative measure of the absolute amount of immobilized JT-95. Because a direct comparison between the presence and absence of BS3 was not included in the present experimental design, the immunoassay results were interpreted as the JT-95-related ELISA signal obtained following BS3-mediated immobilization rather than as a quantitative measure of the specific contribution of BS3. Similarly, because antigen-binding activity was not examined in the present study, the results were interpreted in terms of the immobilization-related ELISA signal rather than as evidence of retained antigen-recognition function after immobilization.

### 4.4. Relationship Among Specific Surface Area, Amino Functionalization, and ELISA Response

In this section, the overall relationships among BET specific surface area, APTES-mediated amino functionalization, and the JT-95-related ELISA signal are discussed by integrating the results obtained from the three particle types.

Across the three particle types, increases in BET specific surface area associated with MACE-induced surface structuring were accompanied by increases in both the ninhydrin response and the JT-95-related ELISA signal. The exploratory bivariate analyses also showed positive associations between BET specific surface area and ninhydrin response (r = 0.89, *p* < 0.0001) and between BET specific surface area and the JT-95-related ELISA signal (r = 0.86, *p* < 0.0001). These consistent trends strongly suggest an association between increased specific surface area, enhanced APTES-mediated amino functionalization, and increased JT-95-related ELISA signal.

Because BET specific surface area was measured once for each particle type and used as the representative value for the corresponding ninhydrin and ELISA measurements, the r and p values obtained from these exploratory analyses should be interpreted in the context of this experimental design. Nevertheless, the consistent increases in the mean ninhydrin response and mean JT-95-related ELISA signal with increasing BET specific surface area support the particle-type-level relationship observed in this study.

The relationships among these parameters were not simply proportional. The BET-normalized ninhydrin results showed that differences in ninhydrin response remained after accounting for specific surface area, suggesting that factors associated with surface morphology may also contribute to amino functionalization. Furthermore, the difference in the JT-95-related ELISA signal between NH-SiNPPs and NH-SiNKPs did not reach statistical significance despite the higher ninhydrin response of NH-SiNKPs, suggesting that factors such as accessibility and spatial arrangement of immobilized IgM may also influence the ELISA signal.

Taken together, the present results support specific surface area as an important factor associated with amino functionalization and the subsequent JT-95-related ELISA signal in surface-structured SiNPs, while also suggesting that surface morphology-related factors may contribute to these relationships. These findings provide a basis for the rational design of SiNP surfaces for antibody immobilization.

## 5. Conclusions

SiNPs with different specific surface areas were prepared by MACE, and the effects of surface modification on APTES-mediated amino functionalization and JT-95-related ELISA signals following BS3-mediated immobilization were investigated. Increasing the specific surface area was associated with enhanced amino functionalization and an increased JT-95-related ELISA signal. Both NH-SiNPPs and NH-SiNKPs exhibited significantly higher JT-95-related ELISA signals than NH-SiNPs. Overall, these findings strongly suggest an association among increased specific surface area, enhanced amino functionalization, and increased JT-95-related ELISA signal, and provide useful design guidelines for SiNP-based antibody immobilization platforms and their future application in immunoassays and biosensing.

## Figures and Tables

**Figure 1:**
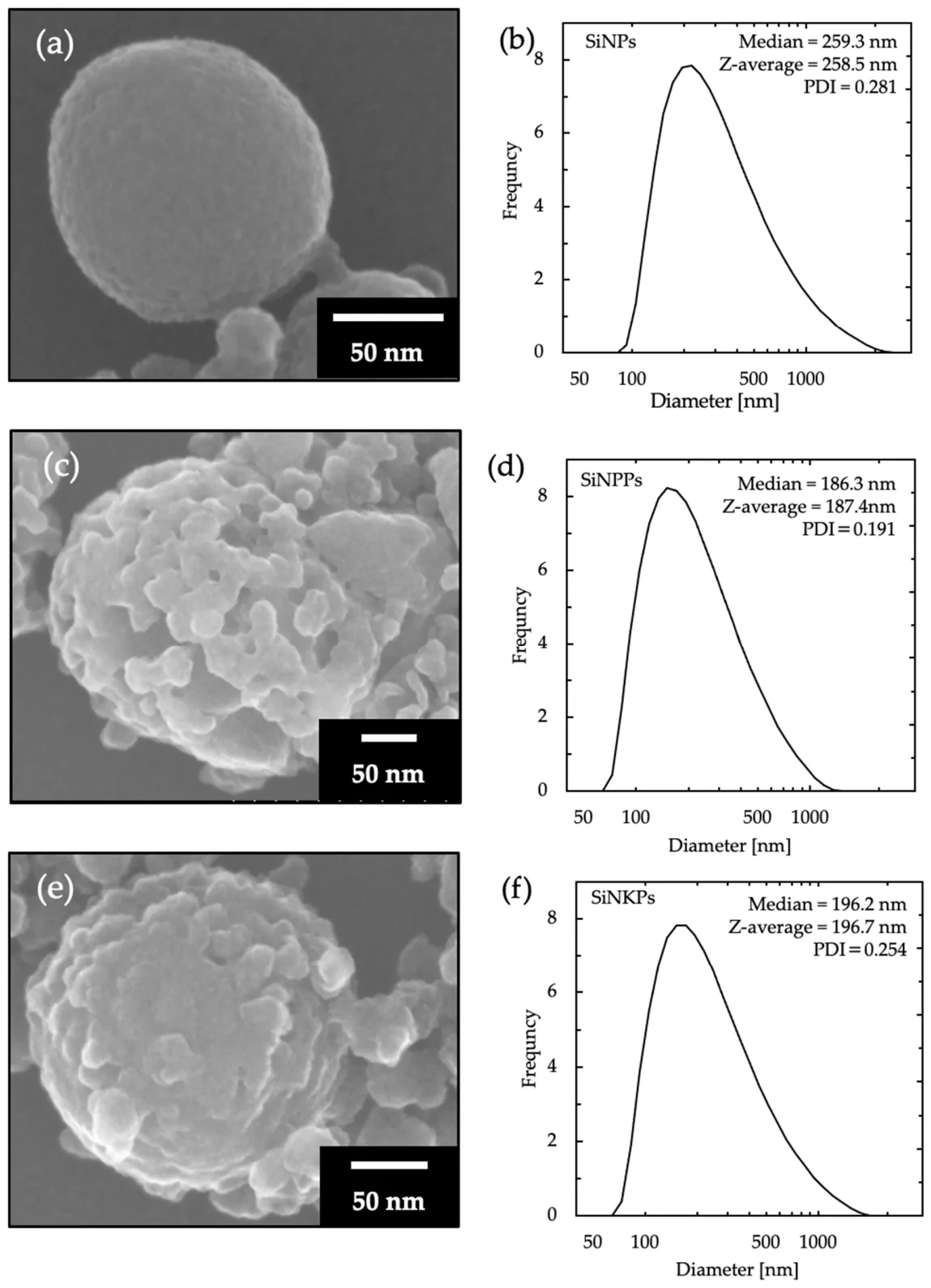
Scanning electron microscopy (SEM) images (**left**) and dynamic light scattering (DLS)-derived hydrodynamic size distributions (**right**) of (**a**,**b**) pristine silicon nanoparticles (SiNPs), (**c**,**d**) porous SiNPs (SiNPPs), and (**e**,**f**) spiky SiNPs (SiNKPs).

**Figure 2:**
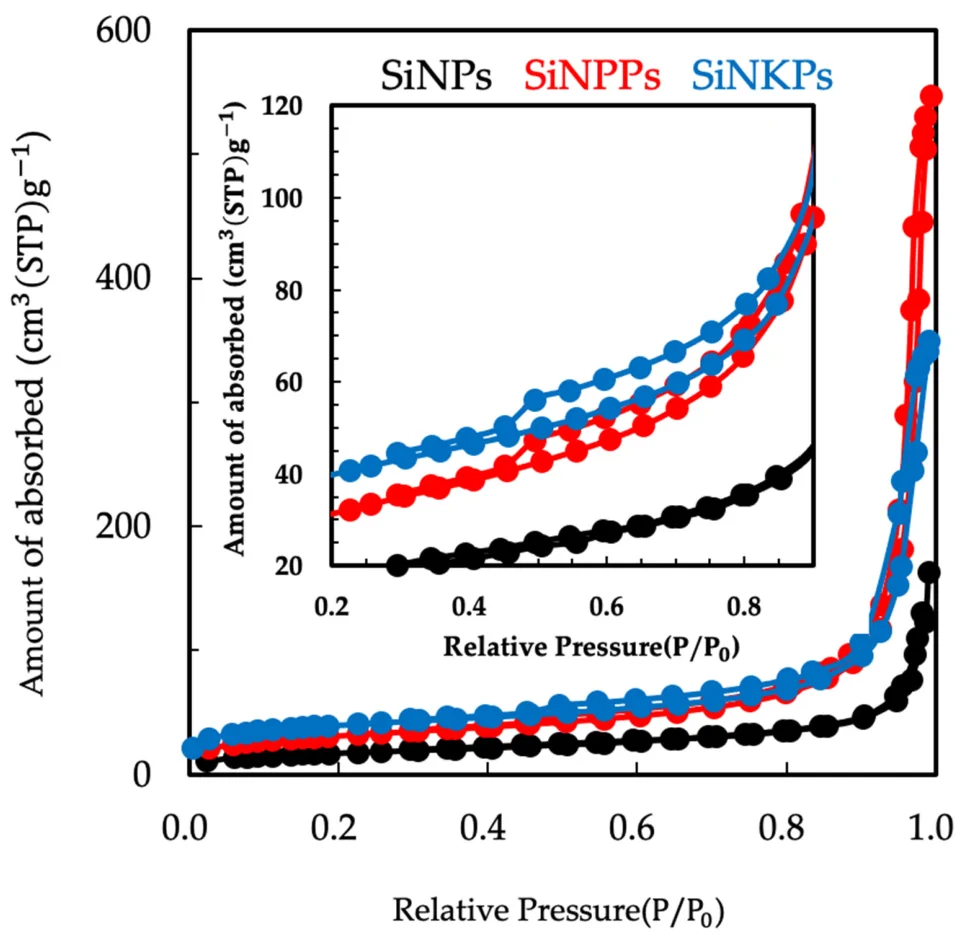
N_2_ adsorption–desorption isotherms of pristine SiNPs, SiNPPs, and SiNKPs. The inset shows an enlarged view of the low-pressure region.

**Figure 3:**
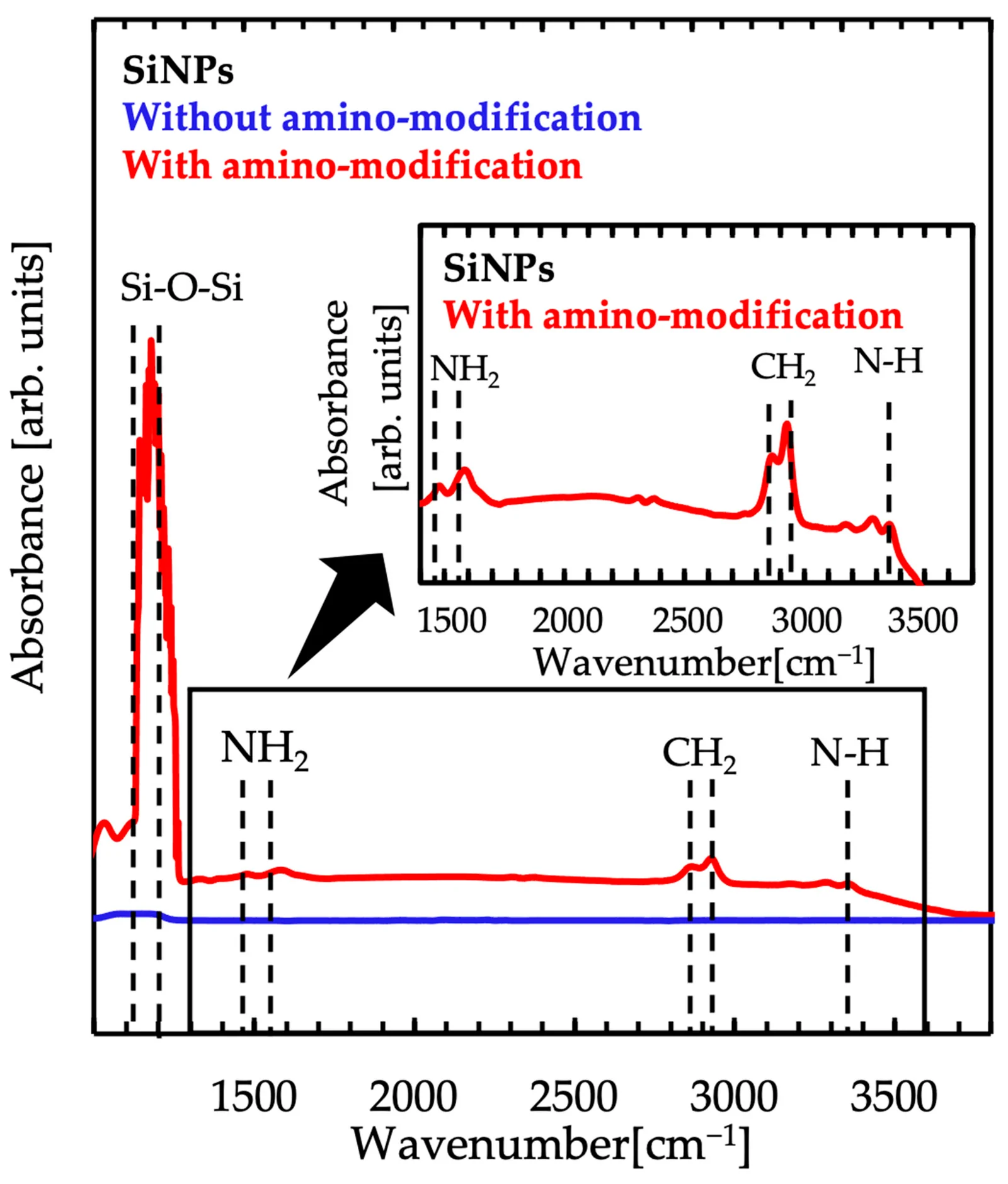
Fourier-transform infrared (FTIR) spectra of SiNPs with amino functionalization (NH-SiNPs, NH-SiNPPs, and NH-SiNKPs; red line) and without amino functionalization (OH-SiNPs; blue line).

**Figure 4:**
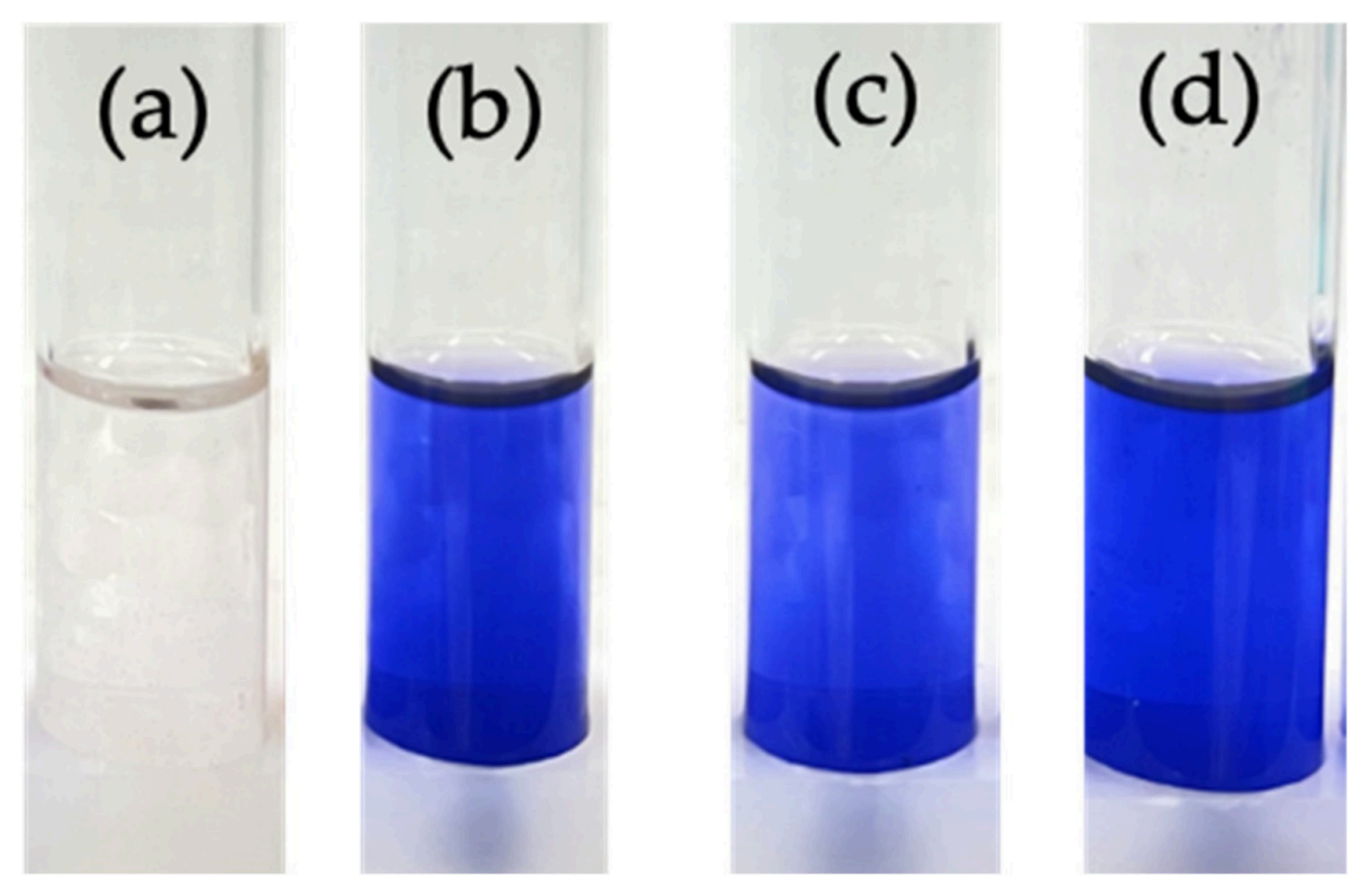
Color development after the ninhydrin reaction for (**a**) OH-SiNPs, (**b**) NH-SiNPs, (**c**) NH-SiNPPs, and (**d**) NH-SiNKPs.

**Figure 5:**
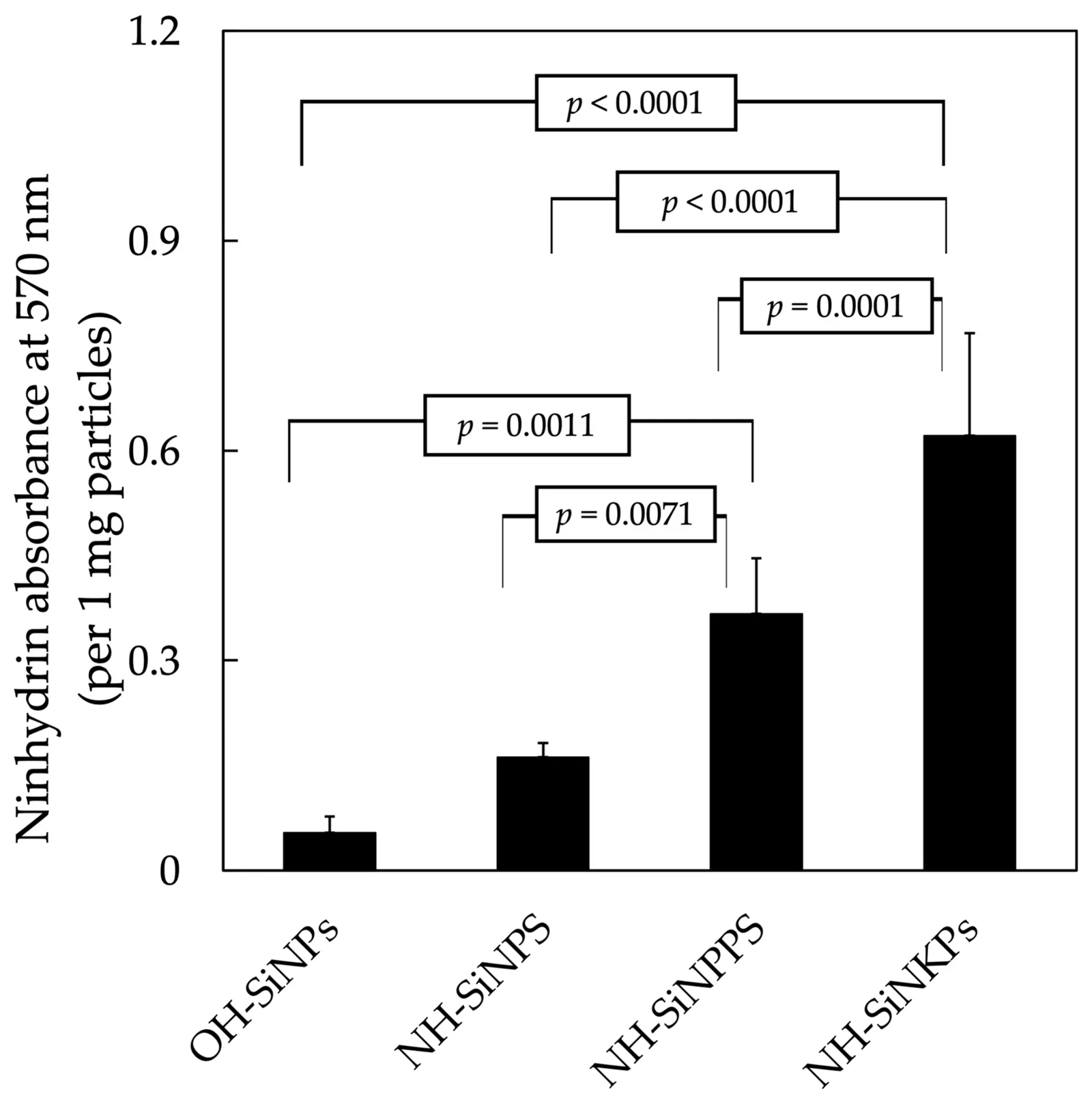
Ninhydrin absorbance at 570 nm normalized to 1 mg of particles for OH-SiNPs, NH-SiNPs, NH-SiNPPs, and NH-SiNKPs. Data are presented as mean ± SD (*n* = 5). Statistical significance was evaluated by one-way ANOVA followed by the Tukey–Kramer multiple-comparison test.

**Figure 6:**
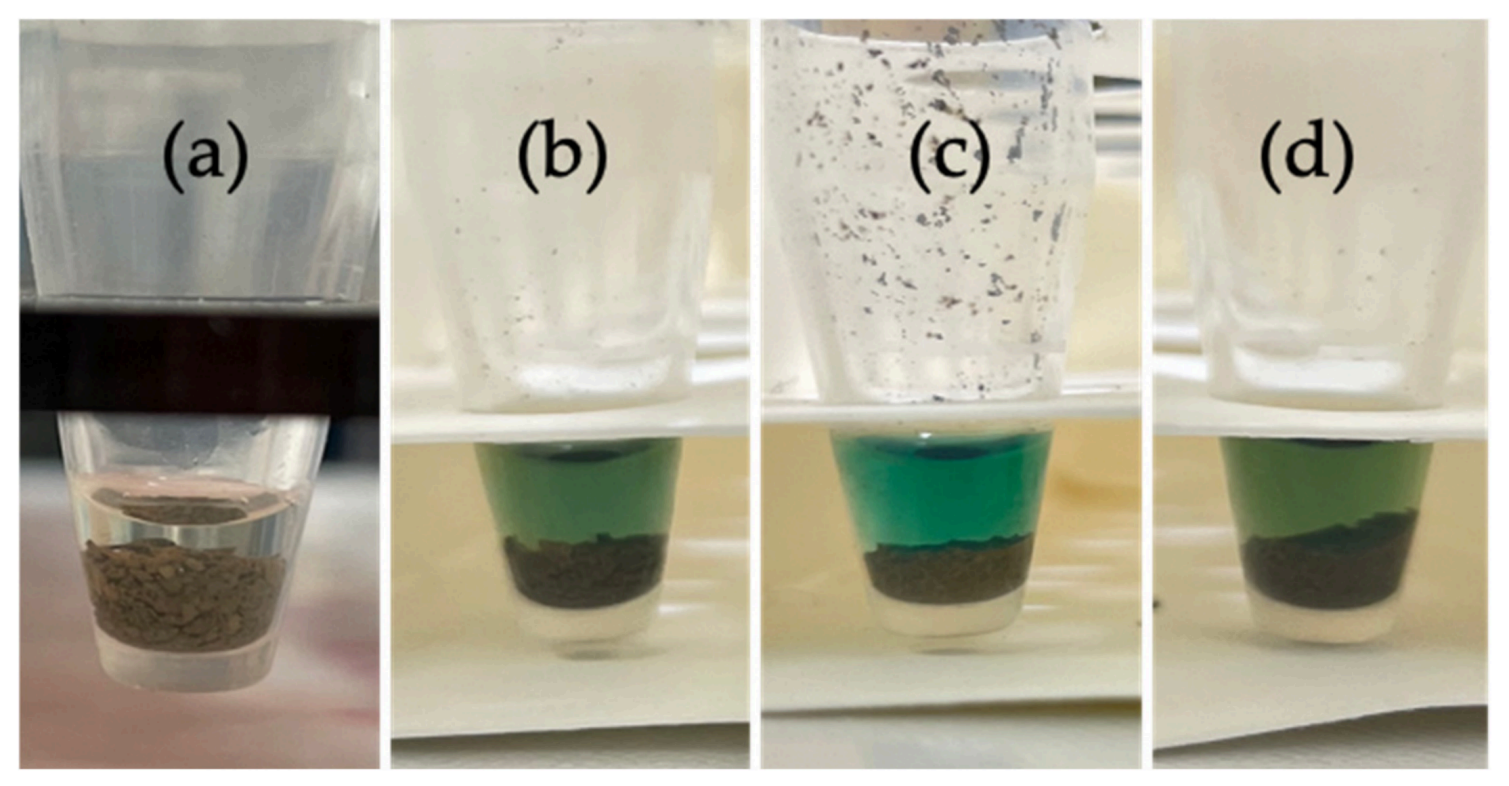
Color development after the ELISA reaction for (**a**) OH-SiNPs, (**b**) NH-SiNPs, (**c**) NH-SiNPPs, and (**d**) NH-SiNKPs.

**Figure 7:**
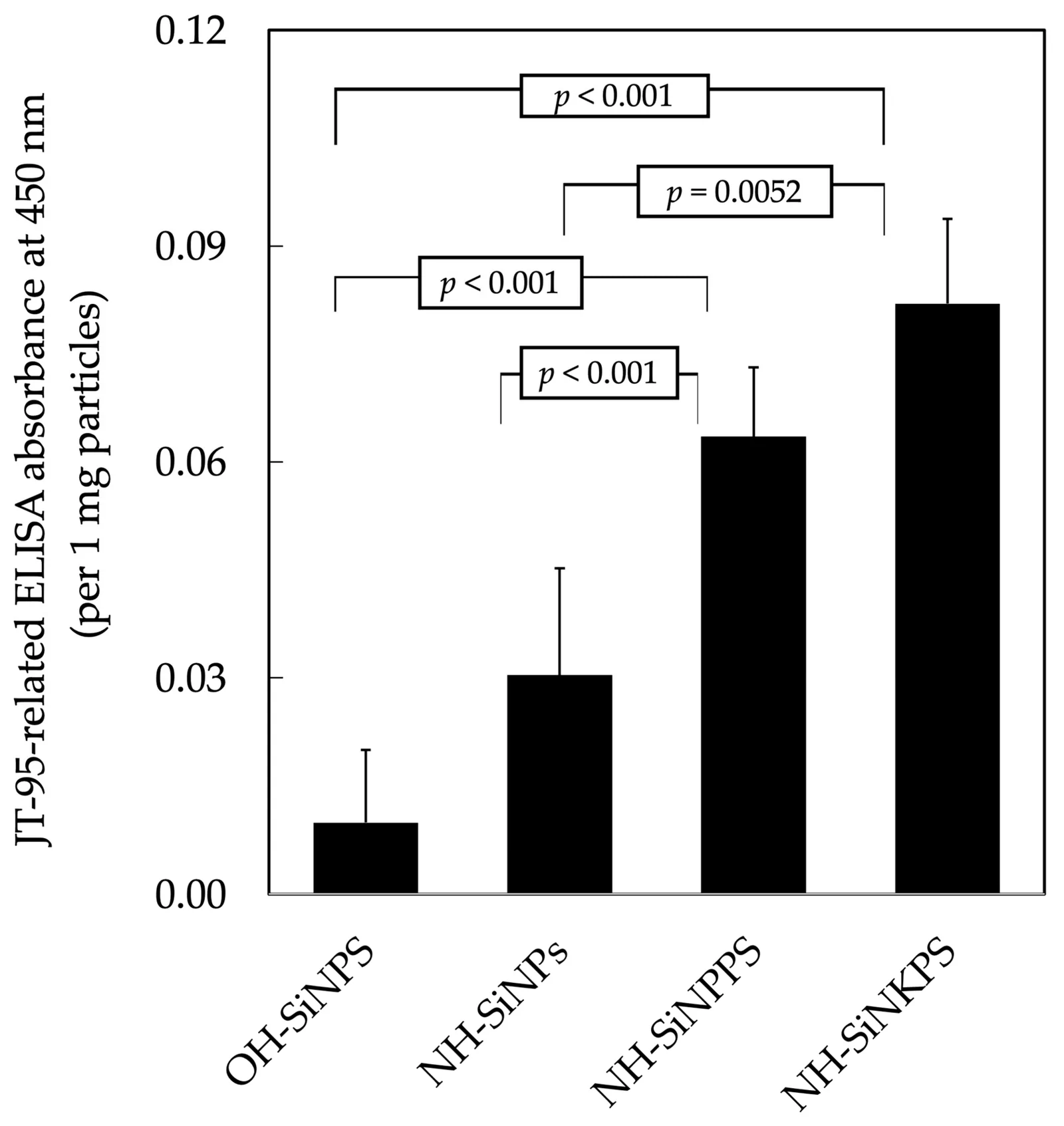
JT-95-related ELISA signal at 450 nm normalized to 1 mg of particles for OH-SiNPs, NH-SiNPs, NH-SiNPPs, and NH-SiNKPs. Data are presented as mean ± SD (*n* = 5). Statistical significance was evaluated by one-way ANOVA followed by the Tukey–Kramer multiple-comparison test.

**Figure 8:**
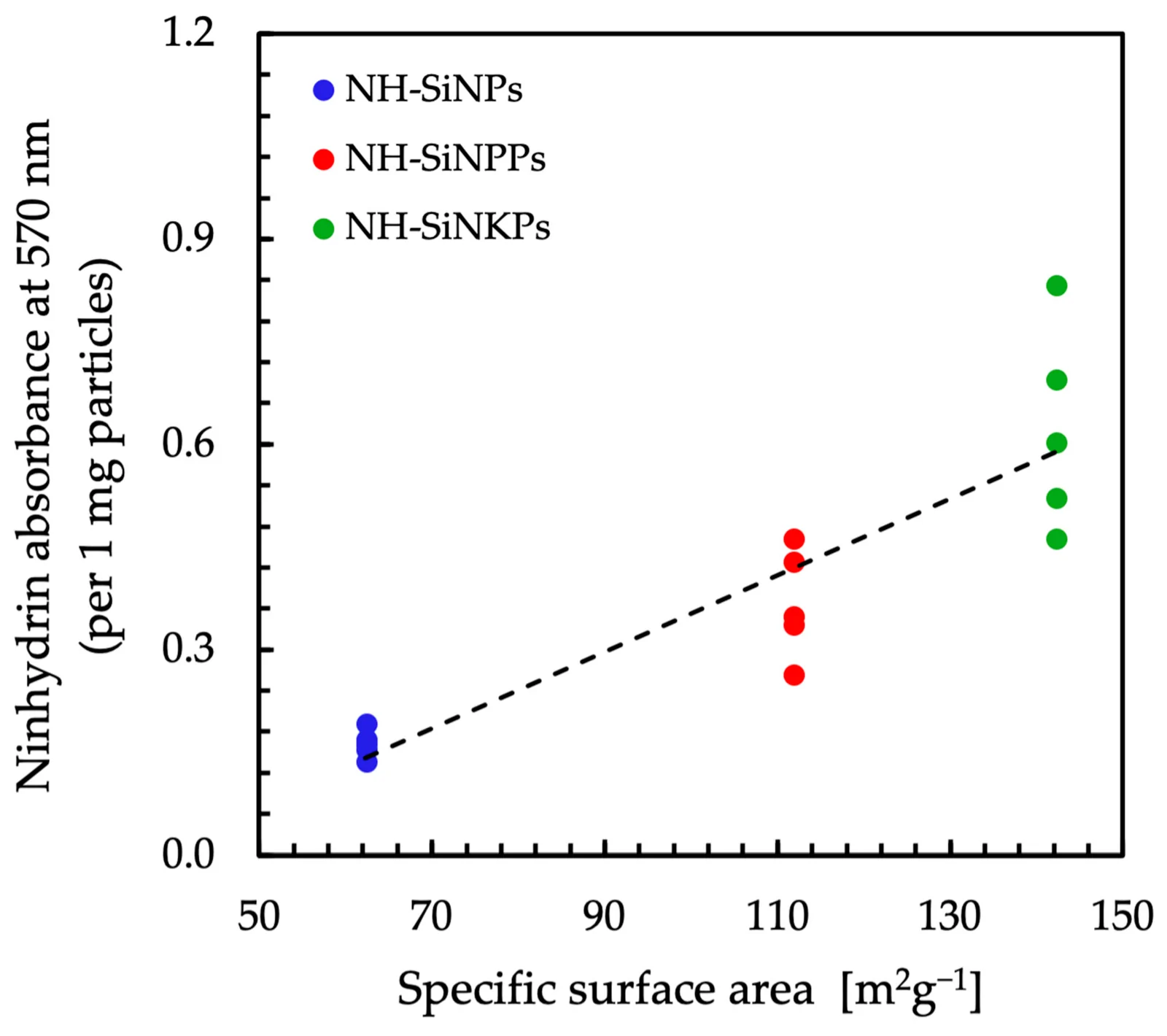
Relationship between BET specific surface area and ninhydrin absorbance at 570 nm normalized to 1 mg of particles. Individual ninhydrin measurements (*n* = 5 for each particle type) are shown as scatter points against the representative BET specific surface area measured for each particle type (*n* = 1 per particle type). The dashed line represents the linear trend.

**Figure 9:**
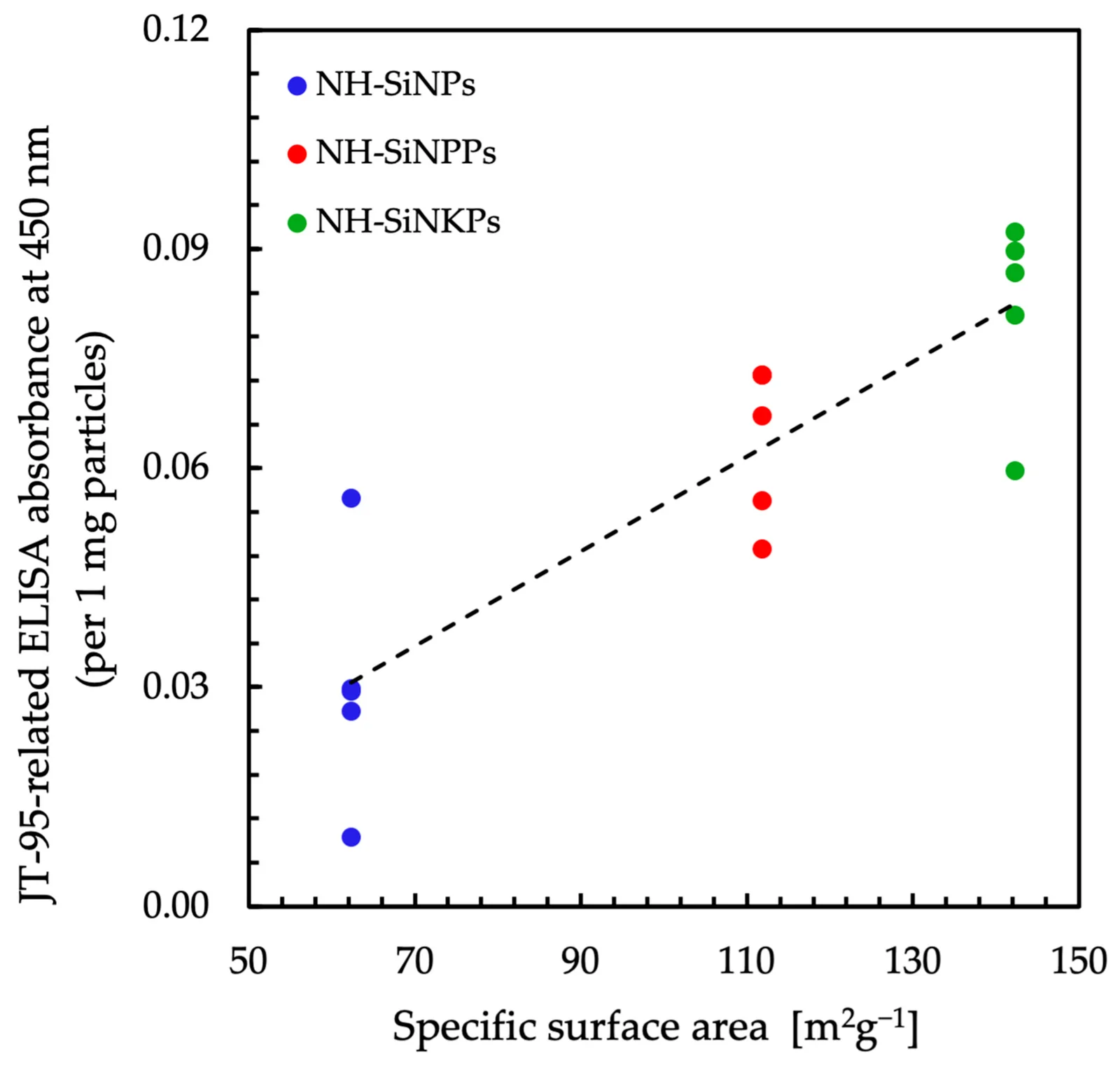
Relationship between BET specific surface area and JT-95-related ELISA signal at 450 nm normalized to 1 mg of particles. Individual ELISA measurements (*n* = 5 for each particle type) are shown as scatter points against the representative BET specific surface area measured for each particle type (*n* = 1 per particle type). The dashed line represents the linear trend.

**Figure 10:**
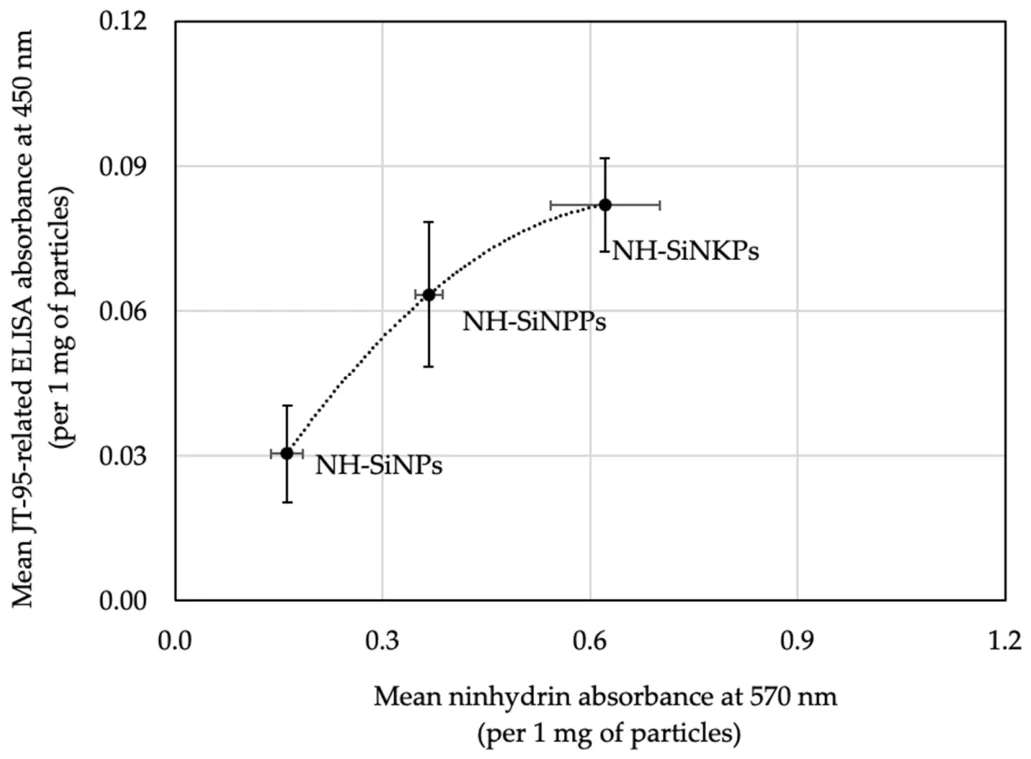
Relationship between mean ninhydrin absorbance at 570 nm and mean JT-95-related ELISA signal at 450 nm for the three particle types. Both values were normalized to 1 mg of particles and represent the mean of five independent measurements (*n* = 5).

**Table 1:** MACE etching conditions used for the preparation of SiNPPs and SiNKPs.

Parameter	SiNPPs	SiNKPs
SiNPs [mg]	300	300
HF [M]	3.0	3.0
AgNO_3_ [M]	0.01	0.02
H_2_O_2_ [M]	0.06	0.06
Final MACE solution volume (mL)	50	50
Etching temperature [°C]	50	50
Etching time [min]	10	10
Stirring speed [rpm]	50	50

Note: The concentrations of HF, AgNO_3_, and H_2_O_2_ represent their final concentrations in the MACE reaction solution.

**Table 2:** Quantitative morphological parameters obtained from SEM image analysis.

Particle Type	Feret Diameter [nm]	MinFeret Diameter [nm]
SiNPs	139.2 ± 35.7	128.4 ± 30.4
SiNPPs	198.2 ± 73.2	175.0 ± 51.7
SiNKPs	247.6 ± 52.9	228.2 ± 43.9

Feret and MinFeret diameters are presented as mean ± SD from five particles obtained from five different SEM images (*n* = 5 particles per particle type).

**Table 3:** Specific surface area of pristine SiNPs, SiNPPs, and SiNKPs.

Materials	Specific Surface Area [m^2^ g^−1^]
SiNPs	62.3
SiNPPs	111.8
SiNKPs	142.2

## Data Availability

The original contributions presented in this study are included in the article. Further inquiries can be directed to the corresponding author.

## Abbreviations

The following abbreviations are used in this manuscript:

APTES	(3-Aminopropyl) triethoxysilane
BET	Brunauer–Emmett–Teller
BJH	Barrett–Joyner–Halenda
BS3	Bis(sulfosuccinimidyl)suberate
DLS	Dynamic light scattering
ELISA	Enzyme-linked immunosorbent assay
FTIR	Fourier transform infrared spectroscopy
HRP	Horseradish peroxidase
JT-95	Monoclonal IgM antibody JT-95
MACE	Metal-assisted chemical etching
PBS	Phosphate-buffered saline
PBS-T	Phosphate-buffered saline containing Tween 20
SiNPs	Silicon nanoparticles
SiNPPs	Porous silicon nanoparticles
SiNKPs	Spiky silicon nanoparticles
TMB	3,3′,5,5′-Tetramethylbenzidine
NH-SiNPs	Amino-functionalized silicon nanoparticles
NH-SiNPPs	Amino-functionalized porous silicon nanoparticles
NH-SiNKPs	Amino-functionalized spiky silicon nanoparticles
OH-SiNPs	Hydroxylated silicon nanoparticles
